# Lessons Learned from Influenza A(H1N1)pdm09 Pandemic Response in Thailand

**DOI:** 10.3201/eid1807.110976

**Published:** 2012-07

**Authors:** Kumnuan Ungchusak, Pathom Sawanpanyalert, Wanna Hanchoworakul, Narumol Sawanpanyalert, Susan A. Maloney, Richard Clive Brown, Maureen Elizabeth Birmingham, Supamit Chusuttiwat

**Affiliations:** Ministry of Public Health, Nonthaburi, Thailand (K. Ungchusak, P. Sawanpanyalert, W. Hanchoworakul, N. Sawanpanyalert, S. Chunsuttiwat);; Thailand Ministry of Public Health–US Centers for Disease Control and Prevention, Nonthaburi (S.A. Maloney);; World Health Organization, New Delhi, India (R.C. Brown); and; World Health Organization, Nonthaburi (M.E. Birmingham)

**Keywords:** national response, surveillance, in-bound screening, influenza-like illness, risk communications, vaccine, pandemic, influenza, Thailand, influenza A(H1N1)pdm09, pH1N1 virus, lessons learned, pandemic planning, viruses

## Abstract

In 2009, Thailand experienced rapid spread of the pandemic influenza A(H1N1)pdm09 virus. The national response came under intense public scrutiny as the number of confirmed cases and associated deaths increased. Thus, during July–December 2009, the Ministry of Public Health and the World Health Organization jointly reviewed the response efforts. The review found that the actions taken were largely appropriate and proportionate to need. However, areas needing improvement were surveillance, laboratory capacity, hospital infection control and surge capacity, coordination and monitoring of guidelines for clinical management and nonpharmaceutical interventions, risk communications, and addressing vulnerabilities of non-Thai displaced and migrant populations. The experience in Thailand may be applicable to other countries and settings, and the lessons learned may help strengthen responses to other pandemics or comparable prolonged public health emergencies.

## Abstract 2

The strengths and weaknesses of this response can inform planning for pandemics and other prolonged public health emergencies.

Cases of influenza A(H1N1)pdm09 virus were first reported to the World Health Organization (WHO) by the US Centers for Disease Control and Prevention (CDC) on April 24, 2009 (*1*). On April 27, the director general of WHO raised the level of the influenza pandemic phase from 3 to 4, and 2 days later, the level was raised to 5 (*2*). In Thailand, because of experience gained during the response to an outbreak of avian influenza A (H5N1) (*3**,**4*), the Ministry of Public Health (MOPH) immediately assumed a central role in coordinating national response efforts to a possible influenza A(H1N1)pdm09 outbreak in that country.

On May 12, 2009, 2 imported cases of A(H1N1)pdm09 virus infection were detected in Thailand, and by the end of the month, 12 more cases were reported by the MOPH. In early June, indigenous outbreaks associated with entertainment centers (*5*), schools (*6*), and military barracks (*7*) were reported. By July, A(H1N1)pdm09 virus transmission was detected in all 76 Thai provinces, and 65 deaths were confirmed to be associated with the infection.

National surveillance data indicated that 2 pandemic waves occurred during the initial 12-month outbreak period. The first wave began in May 2009, peaked in July, and subsided in December; the second wave began in January 2010, peaked in early February, and subsided in April. A third pandemic wave occurred during the latter part of 2010. During 2009–2010, a total of 234,050 influenza cases were reported in Thailand. Of these, 47,433 were laboratory-confirmed to be A(H1N1)pdm09 virus infections; 347 deaths were associated with the confirmed cases (Figure 1).

**Figure 1:**
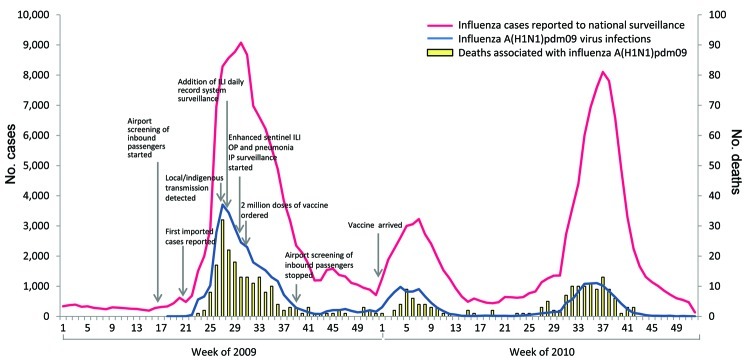
Reported number of influenza cases, laboratory-confirmed influenza A(H1N1)pdm09 virus infections, and deaths associated with confirmed influenza A(H1N1)pdm09 virus infections, Thailand, 2009–2010. ILI, influenza-like illness; OP, outpatient; IP, inpatient.

WHO recommends that countries review their pandemic response and mitigation efforts immediately after a pandemic peak or pandemic phase. In mid-July 2009, the MOPH proposed that the Thai national response be reviewed. This proposal was partially in response to publicly voiced criticism that the pandemic response had not been appropriately handled. To demonstrate transparency and to garner insight from countries that could share valuable insight from their pandemic experience (e.g., Australia and Hong Kong, People’s Republic of China), the Thai MOPH review team was joined by WHO staff and external technical specialists. Seven focus areas were identified for review: 1) surveillance and epidemiology; 2) laboratory services; 3) public health interventions and control measures (including hospital infection control); 4) clinical management; 5) logistics, commodities, and operations; 6) public communications; and 7) measures to assist vulnerable non-Thai populations. The reviews were conducted during August 18–December 6, 2009. In total, 47 team members participated and contributed 271 person-days. Detailed reports and a 28-page summary of the strengths and challenges of the Thai pandemic response were submitted to the minister of health.

The formal review findings (lessons learned) as well as those from a review of the local experience in Thailand are being used to inform current and future pandemic plans in Thailand. They are also likely applicable to other countries and settings and could be used to strengthen responses to future pandemics or to comparable severe, prolonged public health emergencies. In this article, we outline some of the lessons learned during the first 12 months of the national response to the A(H1N1)pdm09 pandemic in Thailand.

## Lessons Learned in Thailand

### Layered Surveillance Is Critical to an Effective Pandemic Response

During the SARS outbreak, the screening of inbound passengers for fever at national/international ports of entry was a common practice by most countries. Thus, politicians and the public believed that the strategy should be included as part of any global epidemic response effort. During the A(H1N1)pdm09 pandemic, this belief created an environment in which it became difficult for the Thai MOPH to target screening activities toward identifying and testing only symptomatic persons arriving from affected countries. At the same time, the MOPH recognized that screening for A(H1N1)pdm09 infection was different than screening for SARS. They realized that SARS-like screening might be of limited value because persons with asymptomatic A(H1N1)pdm09 virus infection could transmit the virus, and persons with symptomatic infection might not have symptoms during inbound border screening. For this reason, fever screening at ports of entry was adopted, not with the expectation of containing early local spread but with the less ambitious aim of possibly detecting infections earlier and slowing the initial spread of virus, thus providing more time to prepare for the pandemic (*8**,**9*). Screening of inbound air passengers to Thailand was implemented on April 27, 2009. Persons with suspected A(H1N1)pdm09 virus infection were treated with antiviral drugs, and close contacts of possible case-patients were given prophylaxis. By June 17, a total of 1,669,501 inbound passengers had been screened at Thailand’s main international airport in Bangkok; 638 of those screened had a fever, and only 2 were confirmed to have A(H1N1)pdm09 virus infection. As the pandemic spread rapidly throughout Thailand, the value of inbound screening was increasingly questioned, and screening was eventually stopped at the end of September 2009.

As expected, despite active screening of inbound air passengers, indigenous transmission and outbreaks were soon observed in entertainment venues and schools. Thailand’s routine national surveillance system includes a national passive notifiable disease surveillance system, which includes notification of pneumonia and influenza cases requiring hospitalization (defined mainly by code criteria of the International Classification of Diseases, Tenth Revision). Thailand’s national influenza surveillance also includes a sentinel system focused on monitoring virus infections; the system includes 8–10 sentinel hospitals that obtain data and specimens from patients seeking medical care for influenza-like illness (ILI). In response to the 2009 pandemic, the MOPH enhanced the surveillance system in 2 ways. First, in May 2009, the MOPH established a daily ILI reporting system to measure geographic and temporal trends for ILI in hospital outpatient departments across the country. Second, the network of sentinel influenza surveillance sites previously established to monitor influenza serotypes was supplemented by an additional 14 new sites. Sites in the expanded network collected respiratory specimens and performed influenza testing for outpatients with ILI and for hospitalized patients with pneumonia. These additions enabled monitoring of spatial-temporal trends and estimations of the prevalence of disease. Overall trends for ILI mirrored those of laboratory-confirmed cases of A(H1N1)pdm09 virus infection, supporting the usefulness of ILI data (Technical Appendix).

In addition to these noted strengths in Thailand’s national surveillance system response, the review team also identified several areas in which improvements should be pursued. These included improving linkages between epidemiologic, laboratory, and clinical data sources; expanding private health care participation in surveillance activities; and strengthening capacity for infectious disease modeling.

### Development of Guidance for Clinical Management and Antiviral Drug Use Is an Iterative Process

Before onset of the pandemic, the Thailand MOPH had established a national stockpile of 300,000 oseltamivir treatment courses, which was sufficient to treat 0.5% of the population. An additional 1 million courses were added when reported cases and deaths appeared to accelerate during the first pandemic wave. The decision to increase the stockpile was prompted in part by the results of local mathematical modeling exercises, which suggested that among the Thai population of 63 million persons, 157,000 could be hospitalized with A(H1N1)pdm09 virus infection and 1,260 could die.

The process of supplying oseltamivir to health facilities was greatly facilitated by a central, Internet-based vendor-managed inventory system, which enabled daily updating of hospital inventories. Individual health care facilities were primarily responsible for monitoring and replenishing their stocks of personal protective equipment, which were supplemented by a network of regional and provincial stockpiles.

**Figure 2:**
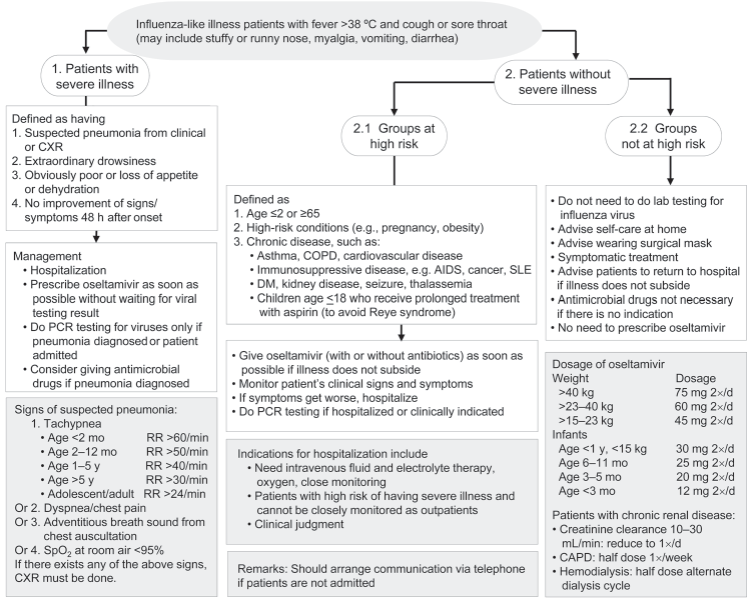
Third edition (July 17, 2009) of clinical practice guidelines for treatment of patients with suspected influenza A(H1N1)pdm09 virus infection in Thailand. The guidelines were prepared by the Clinical Management Taskforce, Thailand Ministry of Public Health, and experts from medical schools. The guidelines are subject to modification according to the pandemic influenza situation; updates are made available at www.moph.go.th. CXR, chest x-ray; COPD, chronic obstructive pulmonary disease; SLE, systemic lupus erythematosus; DM, diabetes mellitus; bid, twice a day; CAPD, continuous ambulatory peritoneal dialysis; RR, respiratory rate; SpO_2_, saturation of peripheral oxygen.

A clinical case management and practice guideline was rapidly made available to all health care workers; the guideline was updated on 3 occasions as new information became available. Revisions focused on the medical management of patients at risk for severe disease, including the need for early administration of oseltamivir (Figure 2). However, anecdotal reports suggested that nationwide adoption of new guidelines by physicians may take up to a month, indicating a need for innovative methods to introduce and implement new guidelines.

An initial policy of screening and testing all persons at risk for A(H1N1)pdm09 virus infection proved problematic. The policy was instituted in an attempt to mitigate spread of infection; however, it led to a widespread public perception that laboratory testing was mandatory for diagnosis and treatment of A(H1N1)pdm09 virus infection. The MOPH subsequently rescinded the policy and issued guidance recommending that persons with suspected A(H1N1)pdm09 virus infection be treated on the basis of clinical rather than laboratory findings. However, patients overlooked the revised policy and continued to request laboratory confirmation of infection, and physicians felt obliged to respond to patient requests; thus, laboratory services became overburdened.

### Laboratory Services Can Become Overburdened

The laboratory system in Thailand provided commendable support to national efforts for combatting A(H1N1)pdm09 virus transmission, especially during the early months of the pandemic. Vast numbers of specimens were tested, and laboratories operated 24 hours a day, 7 days a week. In most cases, laboratory reports were provided within 24 hours of specimen receipt. Laboratory support and expansion were well articulated in Thailand’s national influenza preparedness plan, and surge capacity was quickly organized and implemented.

During the pandemic, the use of PCR was adopted as a standard for laboratory diagnosis of influenza. PCR technology had already been used at the National Influenza Center and 3 university teaching hospitals in Bangkok, and capacity was rapidly established in an additional 14 regional laboratories to share the burden of work and enable rapid testing. The increased capacity for laboratory testing enabled the National Influenza Center and 1 university laboratory to focus on more specialized testing, including molecular sequencing and monitoring of antiviral drug resistance.

In addition to these strengths in the national response effort, the review team also identified several weak areas in need of improvement. The need to strengthen laboratories was highlighted in the national preparedness plan and implemented at the start of the pandemic; however, as discussed above, a national strategy for rational use of laboratory services during high-demand situations was not available to clinicians and public health professionals early in the pandemic. This lack of guidance resulted in an extremely heavy demand for laboratory services. Furthermore, although a plan was in place, it did not anticipate the number of specimens for which testing was requested. During the first 3 months of the pandemic, the National Influenza Center in Thailand processed 10,796 specimens, of which 4,082 were positive for A(H1N1)pdm09 virus infection. Although additional surge capacity was soon developed and implemented, the heavy demands for laboratory testing led to delays in making some results available. Recommendations for future strengthening of the laboratory system included expanding PCR capacity in provincial hospitals, which could serve as referral centers, and clarifying roles specified for each type of laboratory. Such actions would help optimize the laboratory system, maximize efficiency, and enable central laboratories to focus on more specialized functions and research activities.

### Enhanced Infection Control and Surge Capacity Is Needed for Intensive Care Services

During the A(H1N1)pdm09 pandemic in Thailand, several outbreaks were reported among hospital staff and patients (*10*). Infection prevention and control practices appeared to vary at the health care facility level, although most hospitals had dedicated infection control nurses and functional infection prevention and control committees. Before the pandemic, excellent arrangements were in place for screening and triage in hospitals. The arrangements were derived from procedures established during the outbreak of influenza virus A(H5N1), However, despite those arrangements, outpatient services during the first pandemic wave were soon overwhelmed with “worried well” persons seeking information and advice. Hospitals became crowded with patients with ILI, among whom only a small proportion had moderate to severe influenza. Intensive care units in many hospitals became overburdened during the peak of the first wave. Optimal delivery of care might have been achieved through better networking among hospital intensive care units; improved networking could have enabled the sharing of caseloads, resources, and expertise.

### Application and Monitoring of Nonpharmaceutical Interventions Must Be Consistent

The use of good hand hygiene practices, social distancing measures, and face masks was emphasized in national policy and prevention guidelines. These measures were widely promoted and implemented, particularly during the first pandemic wave, when awareness and anxiety levels were high. Implementation of social distancing measures varied by setting, especially in relation to school closure; the varied implementation was probably due in part to the decentralization of decision-making to the local level. The business sector, health foundations, schools, and local community authorities provided good support for public education campaigns. An initial shortage of alcohol gel and face masks was addressed by increasing local production.

An effective mechanism for oversight was not established at the outset of the pandemic, and the lack of such a mechanism presented a challenge for monitoring the effectiveness of public health interventions. Variation and “drift” in the application and implementation of national policies and guidelines were also observed at different administrative levels. Local variations in compliance with national policies and guidelines may have been related to differences in the perception of risk among health professionals and the public and to ineffective communication and feedback systems between authorities at the central level and health providers at peripheral levels. One recommendation for addressing this challenge in the future is to establish a national public health emergency incident command center to coordinate and communicate policies, strategies, and guidance related to an emergency and to monitor their execution and facilitate feedback to concerned parties, particularly on problems related to implementation.

### Availability and Uptake of Pandemic Vaccine Must Be Timely

In July 2009, the Thai government approved the procurement of 2 million doses of A(H1N1)pdm09 vaccine. This amount was determined by using existing registries and other data to estimate the number of health care workers and the number of persons considered to be at high risk for complications related to A(H1N1)pdm09 virus infection. The vaccine arrived at the end of the first wave, during the last week of December 2009, and was targeted to groups at high risk and to frontline health care workers. Vaccination campaigns began in early January 2010, but vaccination uptake was slow and less than projected. There are several possible reasons for this, including the perception of diminishing risk and safety concerns expressed by members of the public, in part related to media reports of (unrelated) fetal deaths in pregnant women who had received the vaccine. Uptake among pregnant women was only 6% (30,000) of the planned 500,000 target population. This percentage is consistent with observations by obstetricians that pregnant women in Thailand were not convinced that the potential benefits of vaccination greatly outweighed any possible risk.

At the same time that Thailand is trying to improve access to pandemic influenza vaccine, it is also trying to establish national capacity for pandemic vaccine production. Since 2008, and with the support of WHO’s Global Action Plan, the Thailand MOPH has embarked on a development project to enhance national capacity for pandemic influenza vaccine development and production. This country project aims to establish capacity for producing inactivated and live-attenuated pandemic vaccines. Although this project did not produce a vaccine in time for the 2009 A(H1N1)pdm09 pandemic, it has served as an excellent platform for further development of the national and regional influenza vaccine capacity in preparation for future pandemics.

### Risk Communication Requires Active Coordination and Monitoring

Public information and risk communication messages were disseminated through a variety of media, including television, radio, and extensively distributed printed materials. However, on several occasions, government officials issued contradictory statements on the status of the pandemic or conflicting health advice. A possible explanation for these shortcomings was the lack of a systematic process to ensure timely delivery of consistent and correct information to the public by politicians, officials, and partners. The communications infrastructure in Thailand is strong; however, some partners could have been better used to assist with disseminating public communications and with collecting feedback regarding the effectiveness of key messages in terms of public understanding and behavior. Furthermore, it would seem intuitive that better communication and public messaging could be used to address the challenges previously discussed in terms of vaccine uptake and health system overloads caused by the worried well. One recommendation proposed addressing these weaknesses by establishing an operational risk communications unit within MOPH.

### Needs of Displaced Persons/Migrants Must Be Included in Pandemic Preparedness and Response Plans

An estimated 144,567 displaced persons live in 9 temporary shelters in Thailand, predominantly on the Thailand/Myanmar border, and ≈2 million registered and unregistered international migrants provide unskilled labor in Thailand. Displaced persons residing in temporary shelters receive basic health care services primarily from nongovernmental organizations, and a compulsory migrant health insurance scheme is in place for registered migrants in Thailand.

Surveillance for influenza and other priority communicable diseases is considered to function well and is linked with the national surveillance mechanisms. However, pandemic response plans for different displaced and migrant population settings were not always congruent, and specific national policies were not completely explicit in defining access to services (care and laboratory diagnostics) and life-saving medicines, such as oseltamivir. In many such settings, surge capacity for delivery of health care was limited, and staff and volunteer health workers were not sufficiently trained in pandemic influenza preparedness and response. Also, public health messages were sometimes not available in the language of displaced or migrant persons (*11*). However, among displaced and migrant populations during the first 12 months of the pandemic, only 1 documented A(H1N1)pdm09 outbreak occurred, with no confirmed deaths, and the review team found that, in general, services were provided on a humanitarian basis when needed.

### External Evaluation of Response Efforts after a Pandemic Peak Is Useful

The team reviewing Thailand’s pandemic response identified numerous strengths and several shortcomings. Because of the timing of the review, some of the lessons learned and some of the shortcomings, particularly in the areas of health care surge capacity, surveillance, and laboratory capacity, were at least partially addressed or rectified during the second and third waves of the A(H1N1)pdm09 pandemic. In addition, the review findings and lessons learned are now being used to guide development of future pandemic preparedness plans in Thailand. The joint MOPH/WHO pandemic review required the mobilization of substantial human and financial resources at a time of already considerable demand. Therefore, consideration should be given to building a strong monitoring and evaluation component into pandemic preparedness plans, including surge capacity for handling review tasks.

## Discussion

We describe lessons learned from the national response to the influenza A(H1N1)pdm09 pandemic in Thailand by reviewing the local experience and a formal MOPH/WHO report on a joint review of the response efforts (*12*). Several of the lessons learned have been identified and discussed in other reports; our work supports and enriches the published data surrounding these lessons.

A report of the WHO Review Committee on the functioning of the international health regulations in relation to the A(H1N1)pdm09 pandemic (*13*) stressed that the response requirements of health care systems needed more attention and strengthening*.* The report also advocated interim case-finding, treatment and management protocols and algorithms, infection control guidelines, guidance on triaging and surge capacity management, and staffing strategies. The findings in the WHO report, much like our findings, emphasized that although avian influenza had made a difference in pandemic preparedness for Asian countries, the 2009 influenza pandemic strained health care and laboratory services, and the strain would have been worse in a more severe pandemic.

Fisher et al., in a review of pandemic response lessons from 6 Asian countries (Singapore, Hong Kong, People’s Republic of China, Malaysia, South Korea, and Vietnam) (*14*), noted some key health challenges similar to those in the Thailand experience: a need to strengthen health care surge capacity (especially intensive care services), an inability of containment measures to prevent cross-border entry of influenza, challenges with the adoption of recommendations for empiric use of oseltamivir, and the insufficient coordination of the dissemination of clinical management and laboratory protocols and updates and other communications. In addition, Hanvoravongchai et al. and the AsiaFluCap Project (*15*) reported results from rapid analyses of pandemic influenza preparedness in 6 Asian countries. Similar to the situation in Thailand, many of the countries were challenged by the need for greater flexibility in pandemic planning and implementation in order to accommodate changing transmission circumstances and different pandemic scenarios.

The importance of a joint review after a pandemic peak or pandemic phase cannot be overemphasized. In Thailand, the review process and reports, which clearly identified strengths and weaknesses of the pandemic response and provided concrete suggestions for how lessons learned might be used to revise plans for dealing with future events, were used to modify the national response during the second and third waves of the 2009 influenza pandemic. In addition, lessons learned from the review are serving as a helpful resource for the development of a new 5-year national strategic plan for preparedness and response to emerging diseases, which will be submitted for official government endorsement in the near future.

## Supplementary Material

Technical Appendix Number of cases of ILI and of confirmed influenza A(H1N1)pdm09 virus infection reported nationally during the first pandemic wave, Thailand, May–July 2009.
