# Supplementary material for: Lessons Learned from Influenza A(H1N1)pdm09 Pandemic Response in Thailand
Source: Emerg Infect Dis. 2012 Jul;18(7):1058–64. doi: 10.3201/eid1807.110976 (PMC3376790; doi:10.3201/eid1807.110976)
Supplement: Technical Appendix — Number of cases of ILI and of confirmed influenza A(H1N1)pdm09 virus infection reported nationally during the first pandemic wave, Thailand, May–July 2009. [file 11-0976-Techapp_1p.pdf]

Article DOI: http://dx.doi.org/10.3201/eid1807.110976

Lessons Learned from Influenza A(H1N1)pdm09 Pandemic Response in Thailand

Technical Appendix

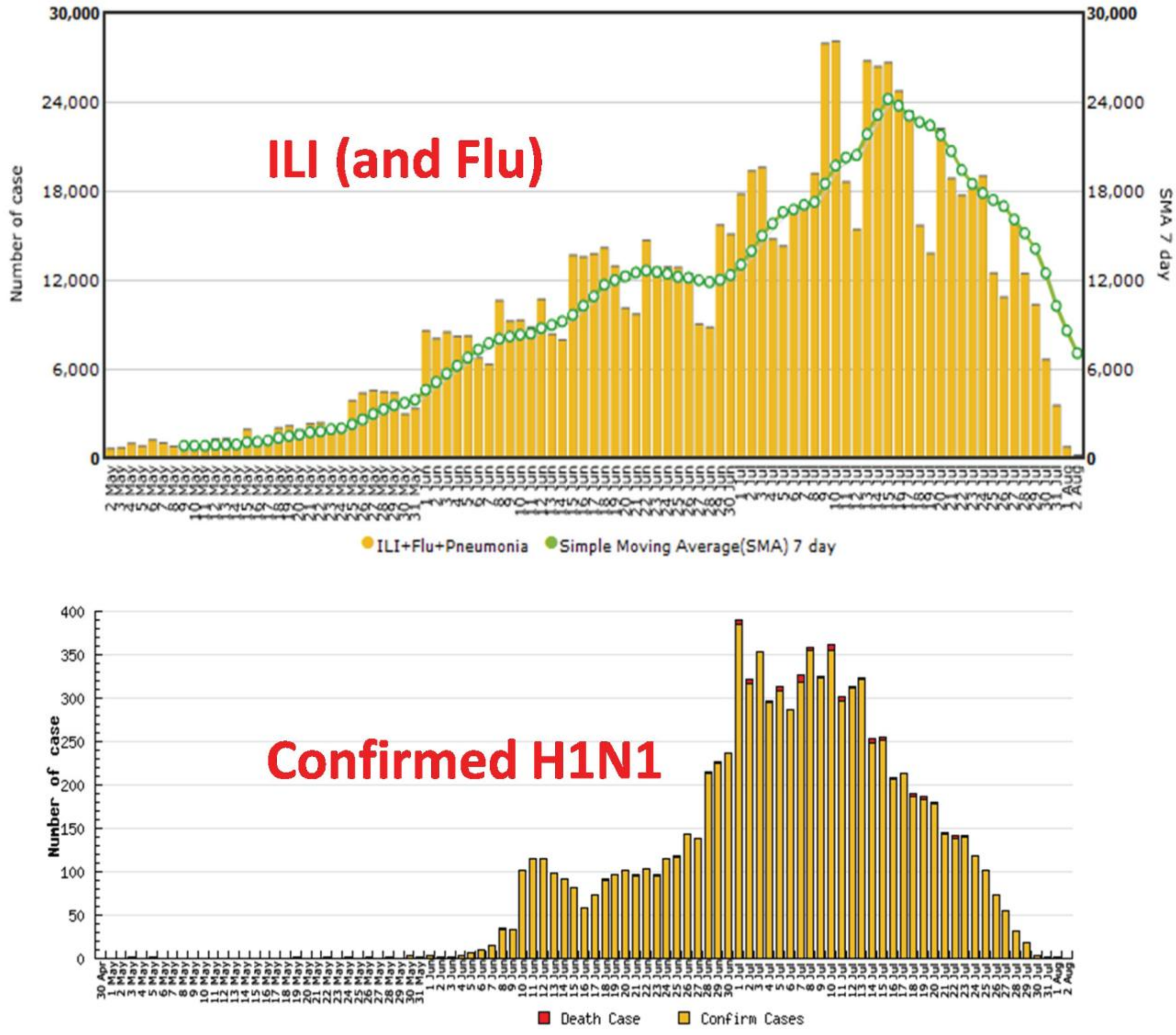

Technical Appendix Figure. Number of cases of ILI and of confirmed influenza A(H1N1)pdm09 virus infection reported nationally during the first pandemic wave, Thailand, May–July 2009. ILI, influenza-like illness; SMA, simple moving average.
